# 16S Based Microbiome Analysis from Healthy Subjects’ Skin Swabs Stored for Different Storage Periods Reveal Phylum to Genus Level Changes

**DOI:** 10.3389/fmicb.2016.02012

**Published:** 2016-12-20

**Authors:** Ingeborg Klymiuk, Isabella Bambach, Vijaykumar Patra, Slave Trajanoski, Peter Wolf

**Affiliations:** ^1^Center for Medical Research, Medical University of GrazGraz, Austria; ^2^Research Unit for Photodermatology, Department of Dermatology, Medical University of GrazGraz, Austria

**Keywords:** storage, skin-swabs, microbiome, standardization, large cohort studies, stability

## Abstract

Microbiome research and improvements in high throughput sequencing technologies revolutionize our current scientific viewpoint. The human associated microbiome is a prominent focus of clinical research. Large cohort studies are often required to investigate the human microbiome composition and its changes in a multitude of human diseases. Reproducible analyses of large cohort samples require standardized protocols in study design, sampling, storage, processing, and data analysis. In particular, the effect of sample storage on actual results is critical for reproducibility. So far, the effect of storage conditions on the results of microbial analysis has been examined for only a few human biological materials (e.g., stool samples). There is a lack of data and information on appropriate storage conditions on other human derived samples, such as skin. Here, we analyzed skin swab samples collected from three different body locations (forearm, V of the chest and back) of eight healthy volunteers. The skin swabs were soaked in sterile buffer and total DNA was isolated after freezing at -80°C for 24 h, 90 or 365 days. Hypervariable regions V1-2 were amplified from total DNA and libraries were sequenced on an Illumina MiSeq desktop sequencer in paired end mode. Data were analyzed using Qiime 1.9.1. Summarizing all body locations per time point, we found no significant differences in alpha diversity and multivariate community analysis among the three time points. Considering body locations separately significant differences in the richness of forearm samples were found between d0 vs. d90 and d90 vs. d365. Significant differences in the relative abundance of major skin genera (*Propionibacterium*, *Streptococcus*, *Bacteroides*, *Corynebacterium*, and *Staphylococcus*) were detected in our samples in *Bacteroides* only among all time points in forearm samples and between d0 vs. d90 and d90 vs. d365 in V of the chest and back samples. Accordingly, significant differences were detected in the ratios of the main phyla *Actinobacteria*, *Firmicutes*, and *Bacteroidetes*: *Actinobacteria* vs. *Bacteroidetes* at d0 vs. d90 (*p*-value = 0.0234), at d0 vs. d365 (*p*-value = 0.0234) and d90 vs. d365 (*p*-value = 0.0234) in forearm samples and at d90 vs. d365 in V of the chest (*p*-value = 0.0234) and back samples (*p*-value = 0.0234). The ratios of *Firmicutes* vs. *Bacteroidetes* showed no significant changes in any of the body locations as well as the ratios of *Actinobacteria* vs. *Firmicutes* at any time point. Studies with larger sample sizes are required to verify our results and determine long term storage effects with regard to specific biological questions.

## Introduction

Published studies on the human derived microbiome, as the entity of all microbial genomes in and on the human body, have increased tremendously in the last decade from 257 publications in 2005 to 5849 in 2015 [retrieved from PubMed by the search term ‘human microbiome’ and updated from Toh and Allen-Vercoe (2015)]. New cost efficient and high throughput next generation sequencing technologies have spurred this scientific development to add a new field with immense significance to medical research (Metzker, 2010). We know that the human microbiome and alterations in the bacterial composition are associated with a wide range of human diseases from neurological [multiple sclerosis (Miyake et al., 2015)], intestinal [Crohn’s disease (Raes, 2016)] and skin (Yu et al., 2015) disorders to infertility (Franasiak and Scott, 2015). Microbiota (the entity of all microorganisms living in and on the human body) may influence our physiology directly by stimulating our immune system, occupying and affecting habitats on the human body defending us against pathogens or influence us through their metabolites (Integrative Hmp (iHMP) Research Network Consortium, 2014). Although the significance of microbiota for human health and physiology is recognized, many studies lack statistical significance due to inter-individual differences of microbiomes and insufficient significant sample sizes for statistical power and calculation of the biological traceability. A prerequisite for processing and analysis of large cohort samples is on the one hand the coordinated collection and on the other hand the reproducible laboratory processing and bioinformatics analysis of samples. For comparison and reproduction of these studies on the human microbiome, standardization of the workflow steps is paramount and already requested from scientific community (Sinha et al., 2015). Future needs to implement microbiome analysis in daily clinical procedures are already drafted (Fricke and Rasko, 2014; Klymiuk et al., 2014). This started process of standardization to increase reproducibility, efficiency and quality of data output on microbiome research projects will not only affect the processing in the wet lab during analyses of sample material. The storage of hundreds to thousands of samples needed for a large cohort study is not only a logistical challenge but requires strict standardization criteria as used for collection, processing, and data analysis. ‘Microbiome’ samples imply a variety of sample materials ranging from stool, swabs, and different body fluids to tissues and biopsies. This variety of samples requires evaluation of their storage conditions and storage time to prevent contamination of biological results from artifacts caused by the experimental procedure (Meisel et al., 2016) and to assure that storage will not alter or destruct valuable information of invaluable samples. Since DNA can degrade through oxidation, hydrolysis, or enzymatic degradation (Gorzelak et al., 2015), we must consider that sampling methods and storage conditions can be a main parameter for microbiome studies and on the data output. These processes must be optimized to reduce DNA degradation and ultimately minimize the variability observed in microbiome analyses. Evaluating the effects of storage temperature, condition (e.g., buffer) and time on the microbiome composition is an important prerequisite for long term storage of microbiome samples in bio banks to utilize this information for personalized medicine approaches (Kinkorova, 2015). The effect of sampling and storage of human derived microbiome samples has already been studied in stool (Roesch et al., 2009; Lauber et al., 2010; Bahl et al., 2012; Carroll et al., 2012; Choo et al., 2015; Gorzelak et al., 2015; Tedjo et al., 2015), vaginal (Bai et al., 2012), sputum (Zhao et al., 2011), and skin (Lauber et al., 2010) specimens. Some previous studies conclude that there were no significant differences in the bacterial community or the richness due to sample storage, although various experimental procedures were used (Zhao et al., 2011; Bai et al., 2012; Carroll et al., 2012; Choo et al., 2015; Tedjo et al., 2015), and most studies conclude that storage at room temperature for several hours or at 4, -20, or -80°C for durations from hours to months did not alter the main biological information (overall community composition and relative abundance of major taxa) on the main habitat specific phyla (Lauber et al., 2010; Carroll et al., 2012; Choo et al., 2015; Tedjo et al., 2015). Lauber et al. (2010) found no significant changes in the phylogenetic diversity of skin samples even after sample storage for up to 14 days at various temperatures, ranging from 20 to -80°C, before DNA isolation. In most setups, statistical separation of samples occurred by test subjects rather than storage conditions. Nevertheless, Bahl et al. (2012) investigated the effect of freezing fecal samples prior to DNA isolation and detected changes in the ratios of some predominant and prevalent phyla. They found that storage of samples at -20°C for about 2 months did not alter DNA yield, but did significantly alter the ratio between *Firmicutes* and *Bacteroides*. Roesch et al. (2009) also found significant differences in the community composition in individual samples following storage at room temperature for 12–72 h before freezing samples at -80°C. Choo et al. (2015) described significant changes due to preliminary treatment and storage at various conditions before final storage at -80°C, though they provide no data on possible long term storage effects. Cardona et al. (2012) found a negative effect on DNA integrity during storage of samples at room temperature or after freezing and defrosting samples before final storage at -80°C. Other studies demonstrated that microbial diversity remains relatively stable among various storage conditions, whereas the relative abundance of main taxa can change dramatically if samples are stored at room temperature for 2 weeks (Cardona et al., 2012). However, all these studies lack information on long term storage effects on microbiome analyses and specifically on human derived skin samples.

In this study, we investigated the bacterial microbial pattern derived from skin swab samples stored for various time periods to provide recommendations for the standardization of storage in long term projects, as already performed for other sample materials (Peakman and Elliott, 2008). Most previous studies demonstrated an influence of storage on the microbial pattern as a function of different freezing conditions prior to long term sample storage due to home self-sampling in most stool based analyses. Definitive conclusions on the influence of long term storage on the results of microbial community in skin samples are still missing. Here, we describe the sampling and analysis of skin swab samples from eight healthy volunteers at three body locations with DNA isolation performed after overnight freezing (d0), 90 days (d90), and 365 days (d365) of storage at -80°C to analyze long term storage effects on the results of bacterial microbial composition. We also discuss other possible sources for alterations that may change or bias the biological results like technical artifacts, such as variability in different lots of nucleic acid isolations kits that should be considered in large scale cohort studies. Our study offers a trendsetting approach for the handling and long term storage of skin microbiome samples and provides valuable information to plan large scale analyses.

## Materials and Methods

### Study Set-up

Skin swab samples were collected from eight healthy volunteers (seven women and one man) between 25 and 60 years in age. None of the volunteers had received antibiotic treatment for 3 months prior to sampling. All sampling procedures were employed for a pilot study of an explorative microbiome project (approved by the Ethics Committee of the Medical University Graz; protocol no. 27-263 ex 14/15). All participants provided informed consent and the study was conducted in accordance with the Declaration of Helsinki.

### Sampling Procedure

Three equivalent samples were taken from each volunteer at three different body locations (forearm exterior left side, V of the chest, and back). Subjects were instructed not to wash or to use any cosmetics on the day of sample collection using skin swabs. Three adjacent quads of 5 cm side length were sampled with a BD Culture SwabsTM EZ Collection and Transport system soaked with sterilized 0.15 M NaCl and 0.1% Tween-20 (Gao et al., 2008). The swabs were cut under sterile conditions into a sterile 1.5 ml reaction tube and were frozen at -80°C immediately after sampling. DNA was extracted after storage at -80°C the day after sampling or after 90 or 365 days of storage before extraction. Unused swabs soaked in the sterile buffer were cut to the collection tubes and used as negative controls for each time point.

### Total DNA Isolation, 16S Library Preparation and Sequencing

Total DNA was isolated from frozen swab samples with a combination of mechanical and enzymatic lysis with the MagnaPure LC DNA Isolation Kit III (Bacteria, Fungi; Roche, Mannheim, Germany) according to manufacturer’s instructions. Three hundred and eighty microliter of bacterial lysis buffer (Roche, Mannheim, Germany) were added directly to the frozen sample and vortexed vigorously for 60 s to ensure bacterial transfer from swabs into solution. Unused swabs and unused buffer tubes without swabs served as negative controls for sampling and DNA isolation. The swabs were removed and the solutions were transferred to Magna Lyser green bead tubes (Roche, Mannheim, Germany), and bead beated for mechanical lysis at 6500 rpm for 30 s two times in a MagNA Lyser Instrument (Roche, Mannheim, Germany). Samples were incubated with 20 μl lysozyme at 37°C for 30 min followed by 30 μl Proteinase K for 1.5 h at 65°C. Enzymes were inactivated at 95°C for 10 min. The remaining steps were performed according to instructions from the Magna Pure DNA isolation kit III (Bacteria, Fungi). Two hundred microliter of each sample were used for DNA purification in a MagnaPure instrument. Total DNA was eluted in 100 μl and stored at -20°C until PCR amplification. For target specific PCR amplification of hypervariable regions the primers 27f (AGAGTTTGATCCTGGCTCAG) and 357r (CTGCTGCCTYCCGTA) were used according to Baker et al. (2003) and synthesized at Eurofins (MWG, Ebersberg, Germany). Five microliter of total DNA extract were used for a 25 μl PCR reaction in triplicates containing 1 x Fast Start High Fidelity Buffer (Roche, Mannheim, Germany), 1.25 U High Fidelity Enzyme (Roche, Mannheim, Germany), 200 μM dNTPs (Roche, Mannheim, Germany), 0.4 μM barcoded primers and PCR-grade water (Roche, Mannheim, Germany). Thermal Cycling was of initial denaturation at 95°C for 3 min, followed by 30 cycles of denaturation at 95°C for 45 s, annealing at 55°C for 45 s and extension at 72°C for 1 min, one cycle of final extension at 72°C for 7 min and a final cooling step to 4°C. Triplicates were pooled, amplification was verified using a 1% agarose gel and 15 μl of pooled PCR product were normalized according to manufacturer’s instructions on a SequalPrep Normalization Plate (Life Technologies, Vienna, Austria). Fifteen microliter of the normalized PCR product were used as the template for indexing PCR in a 50 μl single reaction composed as described for targeted PCR to introduce barcode sequences for each sample according to Kozich et al. (2013). Cycling conditions were the same as for the targeted PCR with only eight cycles for amplification. After indexing, 5 μl of each sample were pooled, 50 μl of the unpurified library were loaded to a 1% agarose gel (Sigma–Aldrich, St. Louis, MO, USA) and then purified from the gel with a Qiaquick Gel Extraction Kit (Qiagen, Hilden, Germany) according to manufacturer’s instructions. The pool was quantified using PicoGreen dsDNA reagent (Life Technologies, Vienna, Austria) according to manufacturer’s instructions and visualized for size validation on an Agilent 2100 Bioanalyzer (Agilent Technologies, Waldbronn, Germany) using a high sensitivity DNA assay according to manufacturer’s instructions. The sequencing library pool was diluted to 4 nM until run on a MiSeqII desktop sequencer (Illumina, Eindhoven, Netherlands). Version 3 600 cycles chemistry (Illumina, Eindhoven, Netherlands) was used according to manufacturer’s instructions to run the 6 pM library with 20% PhiX (Illumina, Eindhoven, Netherlands) and FASTQ files were used for data analysis.

### Data Analysis

In the first data analysis step MiSeq paired-end raw sequence forward and reverse reads were merged using ea-utils v1.1.2 with standard settings, followed by a split library step from the Quantitative Insights Into Microbial Ecology (QIIME, v1.9.1) software. During this step a quality control step removed sequence reads shorter than 200 nucleotides, reads that contained ambiguous bases or reads with an average quality score of <30. Chimera were removed with USEARCH v6.1 method in QIIME against 97% clustered GreenGenes reference 16S rRNA database (v13.8). In the second step, operational taxonomic units (OTUs) picking utilized QIIME open reference pipeline to perform clustering steps at 97% sequence similarity, the taxonomy assignment with UCLUST algorithm, alignment of reference sequences with pyNAST and generation of a phylogenetic tree with FastTree. The OTU table was reduced by removing all OTUs present in only one sample with <10 reads. Prior to rarefaction and subsequent data analysis, the median absolute number of reads was evenly distributed over the storage groups between 138,081 at d0, 142,152 at d90, and 154,087 at d365 (**Supplementary Figure 1A**). To even all samples, we performed a rarefaction to 65,000 sequence reads per sample for further analysis. Downstream data analysis for alpha and beta diversity as well as statistical calculations were performed in R statistical programming language (v3.2.3) using vegan (2.4-0), GUniFrac (1.0), phytools (0.5–38), and phangorn (2.0.4) packages. For alpha diversity analyses, we calculated and compared richness and Shannon diversity index. The effects of different storage conditions were tested with the non-parametric Friedman test, where the normality assumption was violated, followed by a pairwise Wilcoxon signed-rank test, or student’s *t*-test. In case of multiple testing *p*-values were corrected with Benjamini and Hochberg method. Multivariate data analysis of microbiota community dataset was based on two distance measurements Bray–Curtis and weighted UniFrac. UniFrac distances were calculated on a phylogenetic tree and provided a phylogenetic estimate of community similarity, whereas Bray Curtis dissimilarity provided an abundance-weighted measure. Using the generated distance matrices, we visualized the data with the average-linkage agglomerative hierarchical clustering method and Kruskal’s non-metric multidimensional scaling (NMDS). Statistical differences in the overall community composition of samples were assessed using the “permutational manova” test (Adonis in vegan).

## Results

### Skin Swab Samples Overall Analysis

**FIGURE 1:**
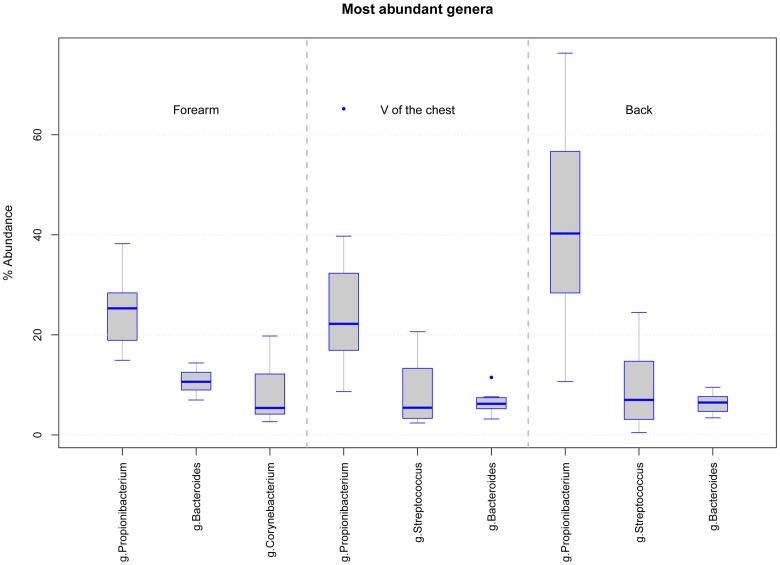
**Boxplot diagram of the relative abundance for the three dominating genera in samples from each body location at d0 (DNA isolation after over-night freezing): forearm samples are dominated by *Propionibacterium*, *Bacteroides*, and *Corynebacterium* and V of the chest and back samples by *Propionibacterium*, *Streptococcus*, and *Bacteroides***.

**FIGURE 2:**
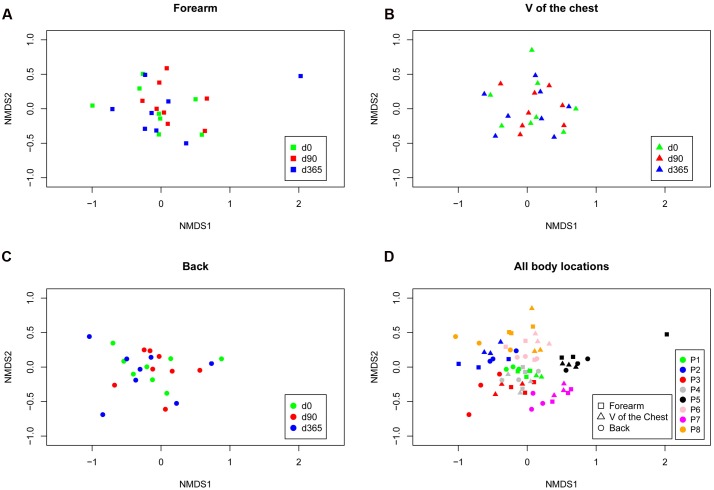
**Non-metric ordination plots based on Bray–Curtis distances for each body location forearm** **(A)**, V of the chest **(B)** and back **(C)** colored according to the storage period and for all locations (separated by symbols) and storage periods combined colored by the volunteer (P1–P8; **D**). Neither calculating each body location individually nor analyzing all locations together revealed a cluster for storage time at -80°C. The clustering occured in inter-individual differences [patient *p*-value = 0.001 (*R*2 = 49%), body location *p*-value = 0.023 (*R*2 = 4.7%), and storage time *p*-value = 0.07 (*R*2 = 4.0%) as analyzed with multivariate permutational MANOVA; Adonis in R.

Skin swab samples from eight healthy volunteers were analyzed from three different body locations and after three sample storage time periods at -80°C. FastQ raw data can be accessed through the SRA accession number SRPO74170 at NCBI Trace Archive. From these 72 specimens and six controls (one for each sample collection date and one for each MagnaPure isolation batch), we analyzed a dataset of 18,136,666 passed filter paired end raw sequence reads (for details in reads distribution see **Supplementary Figures 1A–C**). Depending on the body location, a total number of 5,646 OTUs were detected in forearm samples, 5,058 OTUs in back and 5,901 OTUs in V of the chest specimen. Under reference conditions with DNA isolation after overnight freezing analyzing all samples together, specimen were dominated by the phyla *Actinobacteria* (*M* = 42.5%, *SD* = 19.9), *Firmicutes* (*M* = 30.9%, *SD* = 17.0), and *Bacteroidetes* (*M* = 12.8%, *SD* = 6.9). The most abundant genera in forearm samples were *Propionibacterium* (*M* = 24.9%, *SD* = 10.9), *Bacteroides* (*M* = 12.5%, *SD* = 7.2), and *Corynebacterium* (*M* = 11.2%, *SD* = 14.0). The V of the chest samples contained *Propionibacterium* (*M* = 31.2%, *SD* = 18.8), *Streptococcus* (*M* = 9.9%, *SD* = 7.4), and *Bacteroides* (*M* = 7.3%, *SD* = 3.4). In back samples *Propionibacterium* (*M* = 41.3%, *SD* = 26.5), *Bacteroides* (*M* = 9.2%, *SD* = 7.2), and *Streptococcus* (*M* = 8.6%, *SD* = 8.0) were most abundant (**Figure 1**). The interpersonal differences and the changes among sampled body locations, we observed are well known from large cohort human studies (Turnbaugh et al., 2007; Oh et al., 2013). Non-metric multidimensional scaling (NMDS) of Bray–Curtis distances revealed a clustering of samples based on volunteer (*R*ˆ2 49%, *p*-value = 0.001, **Figure 2D**) followed by body location (*R*ˆ2 4.7%, *p*-value = 0.023, **Figure 2D**). No clustering, however, was found for sample storage duration at -80°C (*R*ˆ2 4.0%, *p*-value = 0.07) calculated for each individual location (**Figures 2A–C**) and for all body locations together (**Figure 2D**). Tree based hierarchical agglomerative clustering with average linkage dendrogram analysis on Bray–Curtis distances accordingly revealed no significant clustering of samples based on the sample storage duration at -80°C but our results show a clustering by volunteer followed by the clustering per body location (**Supplementary Figure 2**).

### Sample Storage Effects on the Results of Microbial Diversity and Richness

**FIGURE 3:**
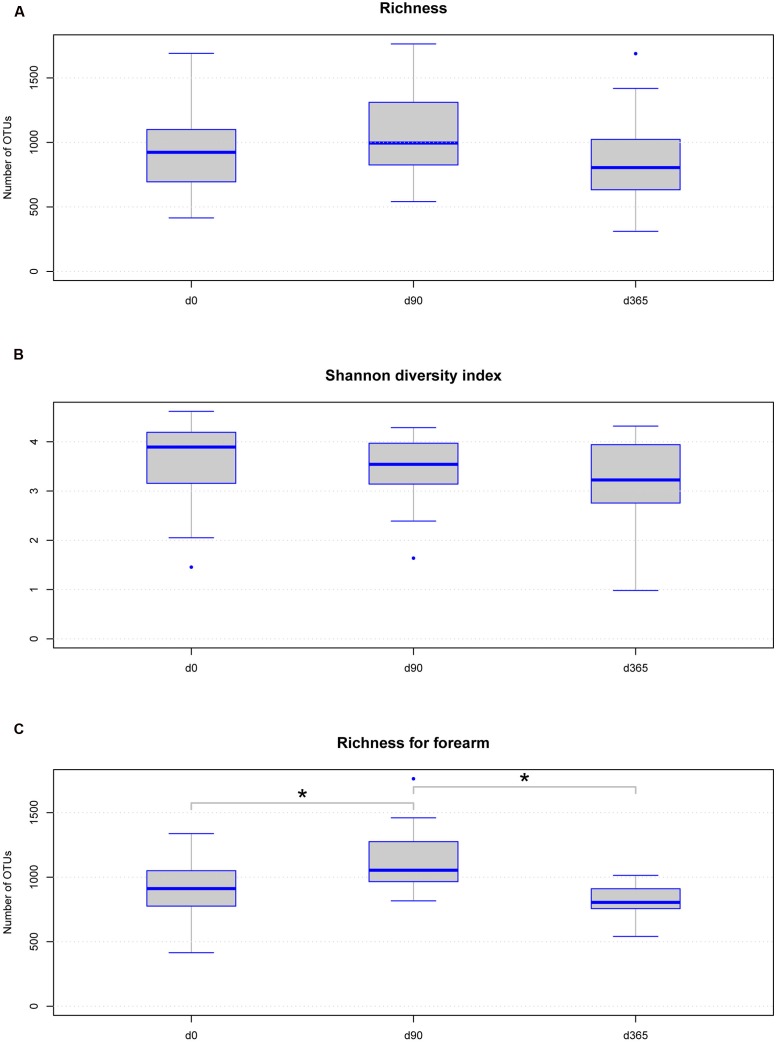
**Boxplot diagram for skin microbial richness** **(A)** and Shannon diversity index **(B)** over all body locations according to sample storage duration: neither richness nor Shannon diversity index is significantly changed among the storage groups. The trend on increased richness at d90 in all samples is not statistically significant. Only the change in forearm sample richness between d0 and d90 (*p*-value = 0.0234) and d90 and d365 (*p*-value = 0.0234) is statistically significant **(C)**, but no difference is observed between d0 and d365 using Wilcoxon test. Significant differences (*p*-value below 0.05) are marked with a line between the affected sample groups and an asterisk.

Under reference conditions, microbial richness of the eight volunteers per body location was between 311 and 1763 OTUs (**Supplementary Table 1**) and the Shannon diversity index was between 0.98 and 4.62 (**Figures 3A,B** and **Supplementary Table 1**). No statistically significant differences were observed in richness and Shannon diversity between the sample groups at different storage durations at -80°C analyzed over all body locations without grouping according to the body location (**Figures 3A,B**). A non-significant trend toward an increase in richness but not in Shannon diversity was observed in the data derived from samples stored at -80°C for 90 days from all body locations. Analyzing different freezing durations per body location separated, the only significant differences in richness were observed between d0/d90 and d90/d365 in forearm samples but not between d0/d365 (**Figure 3C**). No significant differences in Shannon diversity were observed among different freezing periods per body location (**Supplementary Table 1**).

### Sample Storage Effects on the Results of the Ratios of Most Abundant Phyla

**FIGURE 4:**
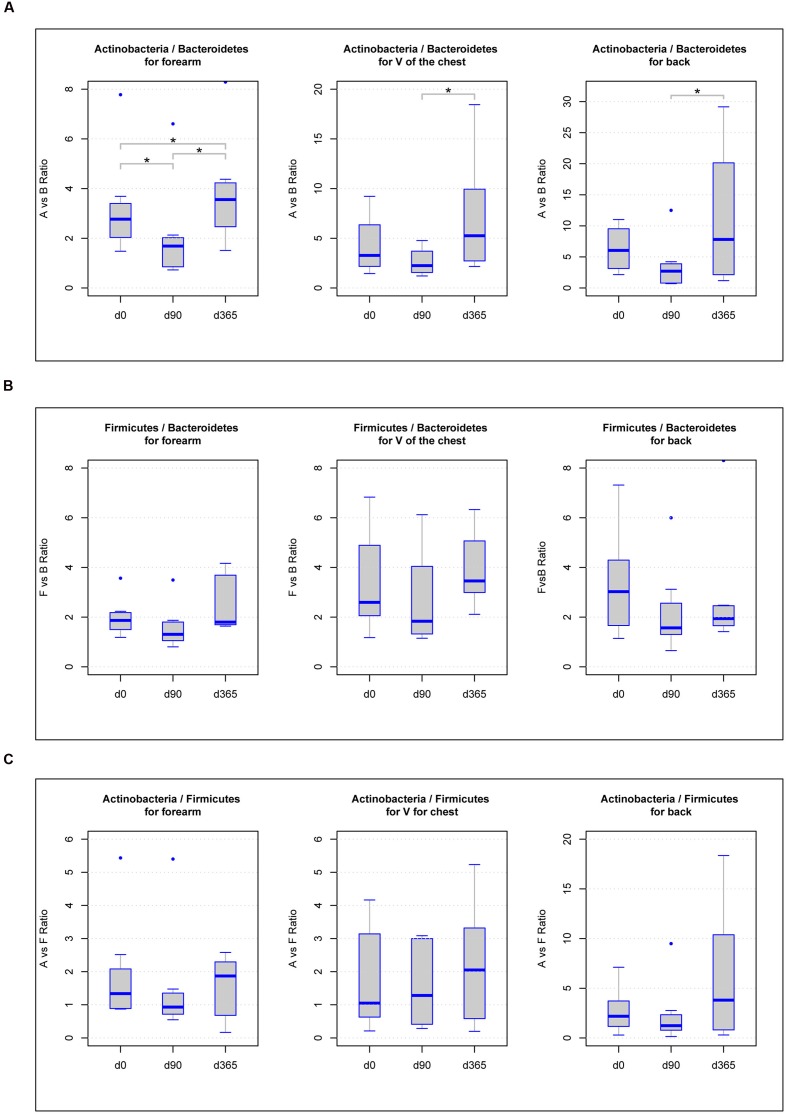
**Boxplot diagram of the ratios of the three main phyla *Actinobacteria* vs. *Bacteroidetes*** **(A)**, *Firmicutes* vs. *Bacteroidetes* **(B)**, and *Actinobacteria* vs. *Firmicutes* **(C)** in forearm, V of the chest and back samples: significant differences in the phyla ratios between storage periods at -80°C (*p*-value below 0.05) are marked with a line between the affected sample groups and an asterisk.

Some former studies discovered differences in the ratios among the main phyla *Actinobacteria*, *Firmicutes*, *Bacteroidetes*, *Proteobacteria*, or *Cyanobacteria* as a function of storage or biological alterations (Stadlbauer et al., 2015; Compare et al., 2016). Accordingly, we analyzed all ratios of the dominant phyla, *Actinobacteria*, *Firmicutes*, and *Bacteroidetes*, found in all samples. Calculating the ratios of *Actinobacteria* vs. *Bacteroidetes* for each body location, we found significant differences (*p*-value < 0.05) in forearm samples across all freezing periods (d0/d90 = 0.0234, d90/d365 = 0.0234, d0/d365 = 0.0234), in V of the chest samples between d90 and d365 (*p*-value = 0.0234) and in back samples comparing d90 and d365 (*p*-value = 0.0234) (**Figure 4A**). For the ratios of *Actinobacteria* vs. *Firmicutes* and *Firmicutes* vs. *Bacteroidetes*, none of the body locations revealed significant differences among any of the three storage periods (**Figures 4B,C**).

### Sample Storage Effects on the Results of Relative Abundance of Main Phyla

**FIGURE 5:**
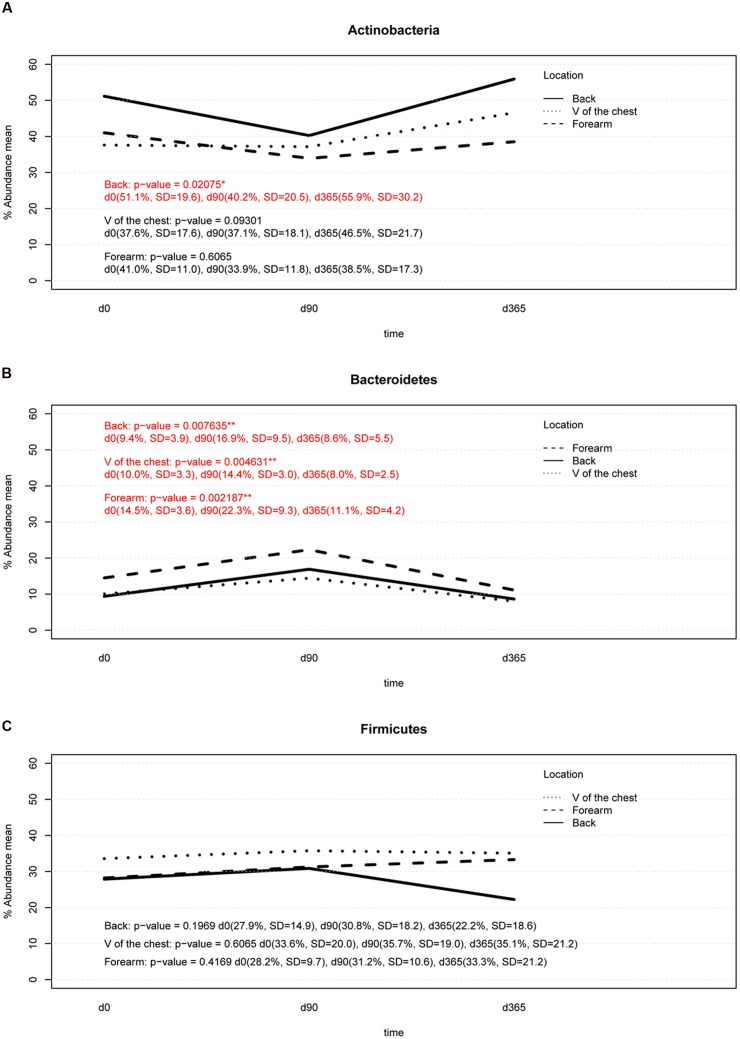
**Two-way interaction plot of the relative abundance for the three most abundant phyla, *Actinobacteria*** **(A)**, *Bacteroidetes* **(B)**, and *Firmicutes* **(C)**: *Actinobacteria* showed a significant change in back samples (*p*-value lower than 0.05) between d90 vs. d365 (*p*-value d0 vs. d90 = 0.0820, *p*-value d90 vs. d365 = 0.0468, *p*-value d0 vs. d365 = 0.5468). The relative abundance of *Bacteroides* increased at d90 in all body locations and returned to baseline at d365 of storage except in forearm samples (d0 vs. d90: *p*-value forearm = 0.0156, *p*-value V of the chest = 0.0351, *p*-value back = 0.0351; d90 vs. d365: *p*-value forearm = 0.0156, *p*-value V of the chest = 0.0234, *p*-value back = 0.0234, d0 vs. d365: *p*-value forearm = 0.0156, *p*-value V of the chest = 0.2500, *p*-value back = 0.4609). No significant changes were detected in *Firmicutes* in any body location.

Summarizing over all analyzed subjects, body locations and storage durations, the most abundant phyla found were *Actinobacteria*, *Firmicutes*, and *Bacteroidetes*. Significant differences (*p*-value < 0.05) in the relative abundance of the phylum *Actinobacteria* was found between DNA isolation after 90 days (d90) and after 365 days (d365; *p*-value = 0.0468) in back samples only (**Figure 5A**). Storage of samples at -80°C from the forearm or V of the chest for 90 or 365 days did not result in significant differences in the relative abundance of this phylum compared to the reference method (DNA isolation after overnight freezing, d0) (**Figure 5A**). The relative abundance of *Bacteroidetes* differed significantly between d0 and d90 in samples from all body locations and between d90 and d365, respectively (**Figure 5B**). Only forearm samples revealed significant differences in the relative abundance of *Bacteroidetes* between d0 and d365 of sample storage (**Figure 5B**). No significant differences were found in the relative abundance of *Firmicutes* in any sampled body location across any storage times (**Figure 5C**).

**FIGURE 6:**
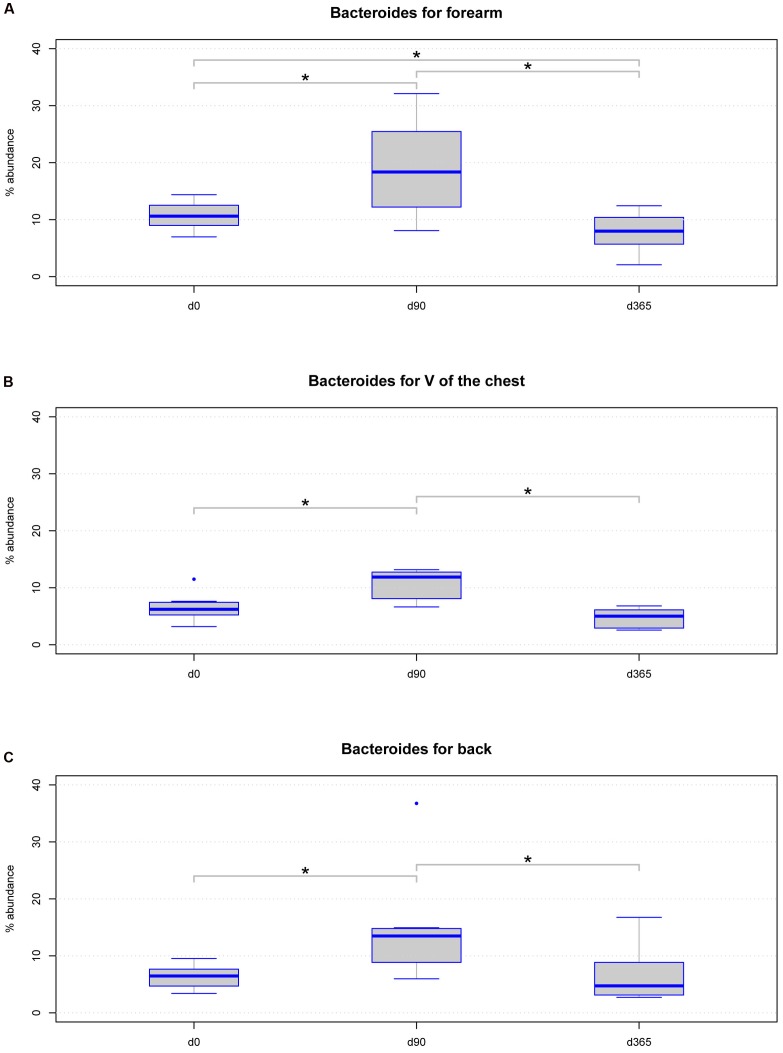
**Boxplot diagram for the relative abundance of the genus *Bacteroides*: this genus is significantly changed in samples from the three body locations** **(A)** forearm (*p*-value = 0.00033), **(B)** V of the chest (*p*-value = 0.00463), and **(C)** back (*p*-value = 0.0098) regarding the storage period using the Friedman statistical test. Significant differences (*p*-value below 0.05) are marked with a line between the affected sample groups and an asterisk.

Further, we analyzed location specific differences across storage periods in a class, order, family, and genus level analysis with Lda Effective Size (LEfSe) (**Supplementary Table 2**). Considering only taxa with a relative abundance of at least 1% in at least 50% of all samples analyzed, we found the genus *Bacteroides* significantly differed in relative abundance in all body locations. LEfSe analysis revealed significant changes in all hierarchical levels the genus *Bacteroides* belong to **Supplementary Table 2**. Further analysis on *Bacteroides* revealed the genus significantly altered between d0 and d90 and between d90 and d365 among all body locations (**Figures 6A–C**). However, between d0 and d365 significant differences for *Bacteroides* were only detected in forearm samples but not in V of the chest and back samples (**Figures 6A–C**).

## Discussion

We analyzed the effects of long term storage at -80°C from skin derived dermal microbiome swab samples on the results of microbial composition. Previous studies investigated the effect of sample storage at different temperatures before final storage at -80°C for weeks primarily in human stool samples, which found minimal to no effects on the analyzed microbial patterns. Our study on eight healthy volunteers investigated for the first time the effect of long term storage on skin derived microbiome samples for up to 1 year at recommended conditions on the data outcome. The three most dominant genera found in our forearm (*Propionibacterium*, *Bacteroides*, *Corynebacterium*), V of the Chest (*Propionibacterium*, *Streptococcus*, *Bacteroides*) and back samples (*Propionibacterium*, *Streptococcus*, *Bacteroides*) under reference conditions at d0 correspond to genera found in former studies on the human skin microbiome (Grice et al., 2008). The estimated microbial richness and the Shannon diversity index were within expected values compared to other skin studies (Grice et al., 2008; Zeeuwen et al., 2012; Meisel et al., 2016). Analyzing our storage groups over all body locations together, no significant differences were found in the richness and Shannon diversity index among sample groups of different storage periods or in the number of observed OTUs. There was an insignificant tendency for increased richness, but not Shannon diversity, at d90 (**Figures 3A,B**). These results correspond to former studies on different storage conditions of stool samples that do not find statistically significant differences in microbial richness due to different storage preconditions (Bai et al., 2012; Flores et al., 2015; Tedjo et al., 2015). The high stability of DNA even after microbial organism death and the robustness of the 16S rRNA PCR based microbiome analysis method may account for this persistent finding. Nevertheless, analyzing the storage groups separated per body locations a significant change in the richness of forearm samples was detected between d0 vs. d90 and d90 vs. d365 but in no other body location. In our study the main biological parameters remained valid between storage groups and samples clustered according to volunteer and body location rather than storage time (**Figures 2A–D**). However, some of the dominant phyla and genera detected in the different skin locations of the eight volunteers showed significant changes over storage periods. The phylum *Actinobacteria* changed significantly between d90 vs. d365 in back samples only and the phylum *Bacteroidetes* between d0 vs. d90 and d90 vs. d365 in all body locations (**Figure 5**). Between d0 and d365 the phylum changed only significantly in forearm samples. Additionally the genus *Bacteroides* showed significant changes in all body locations (**Figure 6**) between d0 vs. d90 and d90 vs. d365. Changes between d0 and d365 are only statistically significant in forearm samples.

Analyzing the ratios of the most abundant phyla detected in the skin swab samples (*Actinobacteria*, *Bacteroidetes*, and *Firmicutes*) revealed statistically significant differences in *Actinobacteria* vs. *Bacteroidetes* across storage durations. In all body locations, differences were found between d90 and d365 and also in forearm samples between d0 and d90 and d0 and d365, respectively (**Figure 4A**). These results may indicate biological changes but also possible technical artifacts caused by different DNA isolation kit batches used at d0, d90, and d365. As such, differences in the relative abundance of *Actinobacteria* between d90 and d365 were only detected in back samples and of *Bacteroidetes* were observed between d0 and d90 and between d90 and d365 in all body locations but the change between d0 and d365 only in forearm samples (**Figures 5A,B**). No differences in the relative abundance of *Firmicutes* were observed among the three time points at all. The reasons for changes in the relative abundance of phyla may occur from the different cell wall characteristics of gram-negative and gram-positive bacteria. *Firmicutes* and *Actinobacteria* are gram-positive organisms probably less affected by DNA degradation due to destruction of the microbial cell wall during storage. In contrast, *Bacteroidetes* belong to the group of gram-negative bacteria that may be more affected by cell death caused DNA degradation by, e.g., oxygen and enzymes. Using the LefSeq and Friedmann test analyses, the only genus differentially abundant across the storage groups was the gram-negative genus *Bacteroides* (**Figures 6A–C**). However, our data does not support this hypothesis of alterations due to DNA degradation as the relative abundance of *Bacteroides* increased by d90 and decreased to reference baseline levels at day 365 (except in forearm samples). This pattern may indicate a technical issue that should be considered in the design of large scale studies although the observed changes between d0 and d365 in forearm samples cannot be fully explained by technical issues. The influence of DNA storage at -20°C until PCR amplification should also be kept in mind. However, one would expect constant DNA degradation and impaired PCR amplification rather than a change in the relative abundance or ratios of dominating phyla and genera.

To answer the fundamental and pressing questions in microbiome research and their relation to human health, large cohort studies will provide further reliable scientific and statistically valid results. Sample size, storage conditions and quality, as well as data analysis, must be appropriate and standardized. This is particularly important for large cohort studies in which sampling and storing microbiome specimens for several years is necessary. In these studies the effect of storage time and inherent variability in DNA extraction batches, as well as library preparation and sequencing, needs to be reconsidered to ensure reproducibility and standardization across studies and in clinical treatment applications. Standardization of sample storage procedures with instant flash-freezing and continuous evaluation through calibration samples throughout the total project duration should be mandated.

To overcome the difficulty inherent in microbiome specimen storage durations, immediate nucleic acid extraction may seem to be advantageous. Our results may indicate that the risk to create a technical bias through different lots of DNA isolation kits may be higher than the bias found in different sample storage times at recommended conditions (-80°C or lower) especially for large cohort skin microbiome studies. In addition, it is not recommended to change the nucleic acid isolation technique to keep samples matchable once a method has been established. While the extracted DNA can be stored at -20°C or even at higher temperatures for long periods of time, it can be exposed to additional sources of variability through degradation induced by enzymatic processes, oxygen degradation or repetitive freeze-thaw cycles, which can bias the results.

Collection of well accepted specimens, such as skin swabs, is critical for future research endeavors. Our study provides a critical reference to elucidate the storage periods for skin microbiome studies. The main biological endpoints and parameters (clustering of samples according to volunteers and body locations but not to storage time points) used for analysis after different sample storage times remained valid throughout our study. Nevertheless, we observed changes in the ratios of the most abundant phyla (*Actinobacteria* vs. *Bacteroidetes*), in the relative abundances of the most abundant phyla (minor changes in *Actinobacteria*, distinct changes in *Bacteroidetes*), in the relative abundance of the genus *Bacteroides* as well as in the richness of forearm samples among sample groups of different storage periods. Some of these results should be interpreted with caution as, we cannot rule out a technical issue by the use of another batch of DNA isolation kit at d90. Another limitation of our study is the limited sample size and gender imbalance (with female predominance of participants due to the availability of volunteers). Studies with larger sample sizes are needed to confirm our results with regard on the potential influence of long term storage effects of specimens on specific biological endpoints.

## Author Contributions

IK: Study design, analyzed the data, and wrote the manuscript. IB: Study design and performed experiments. ST: Analyzed the data, prepared the figures, and revised and wrote the manuscript. VP: Revised and wrote the manuscript. PW: Study design and revised and wrote the manuscript.

## Conflict of Interest Statement

The authors declare that the research was conducted in the absence of any commercial or financial relationships that could be construed as a potential conflict of interest.
