# Supplementary material for: 16S Based Microbiome Analysis from Healthy Subjects’ Skin Swabs Stored for Different Storage Periods Reveal Phylum to Genus Level Changes
Source: Front Microbiol. 2016 Dec 20;7:2012. doi: 10.3389/fmicb.2016.02012 (PMC5167739; doi:10.3389/fmicb.2016.02012)
Supplement: Supplementary file 3 [file Image_1.PDF]

frontiers

Supplementary Material

16S based microbiome analysis from healthy subjects' skin swabs stored for different storage periods reveal phylum to genus level changes

Ingeborg Klymiuk[1*], Isabella Bambach[2], Vijaykumar Patra[2], Slave Trajanoski[1], Peter Wolf[2]

*** Correspondence:** Corresponding Author: ingeborg.klymiuk@medunigraz.at

1 Supplementary Figures and Tables

1.1 Supplementary Tables

Supplementary Table 1: Richness and Shannon alpha diversity descriptive statistics summary for all body locations and storage periods.

Supplementary Table 2: LEfSe analysis on genus, family, order and class levels for each body location.

1.2 Supplementary Figures

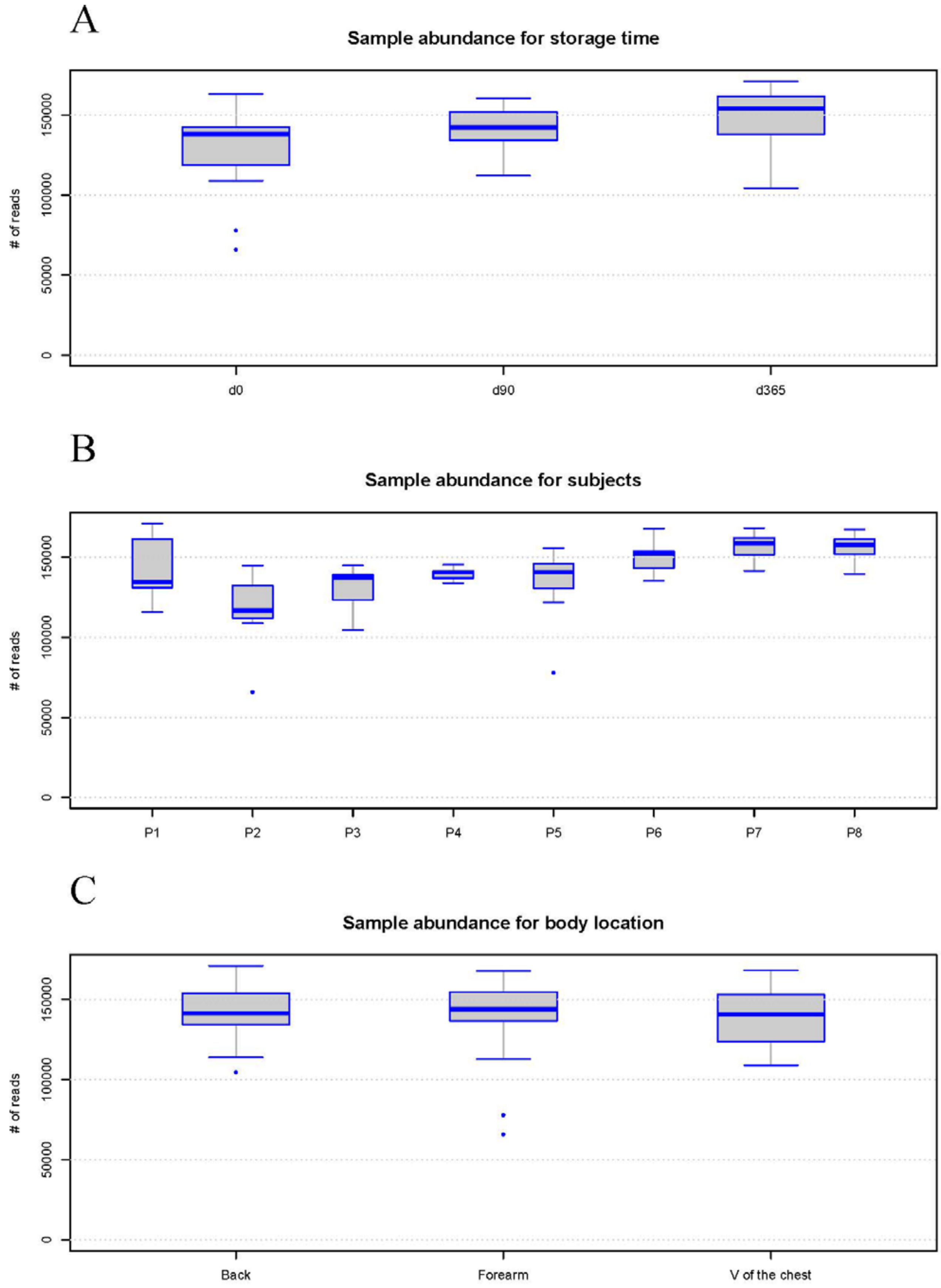
A
Sample abundance for storage time
# of reads
0
50000
100000
150000
d0
d90
d365
B
Sample abundance for subjects
# of reads
0
50000
100000
150000
P1
P2
P3
P4
P5
P6
P7
P8
C
Sample abundance for body location
# of reads
0
50000
100000
150000
Back
Forearm
V of the chest

Supplementary Figure 1.
Boxplot diagrams of the distribution of read numbers grouped by (A) storage time, (B) volunteer (P1 to P8) and (C) body location, demonstrating the constant and comparably reads distribution.

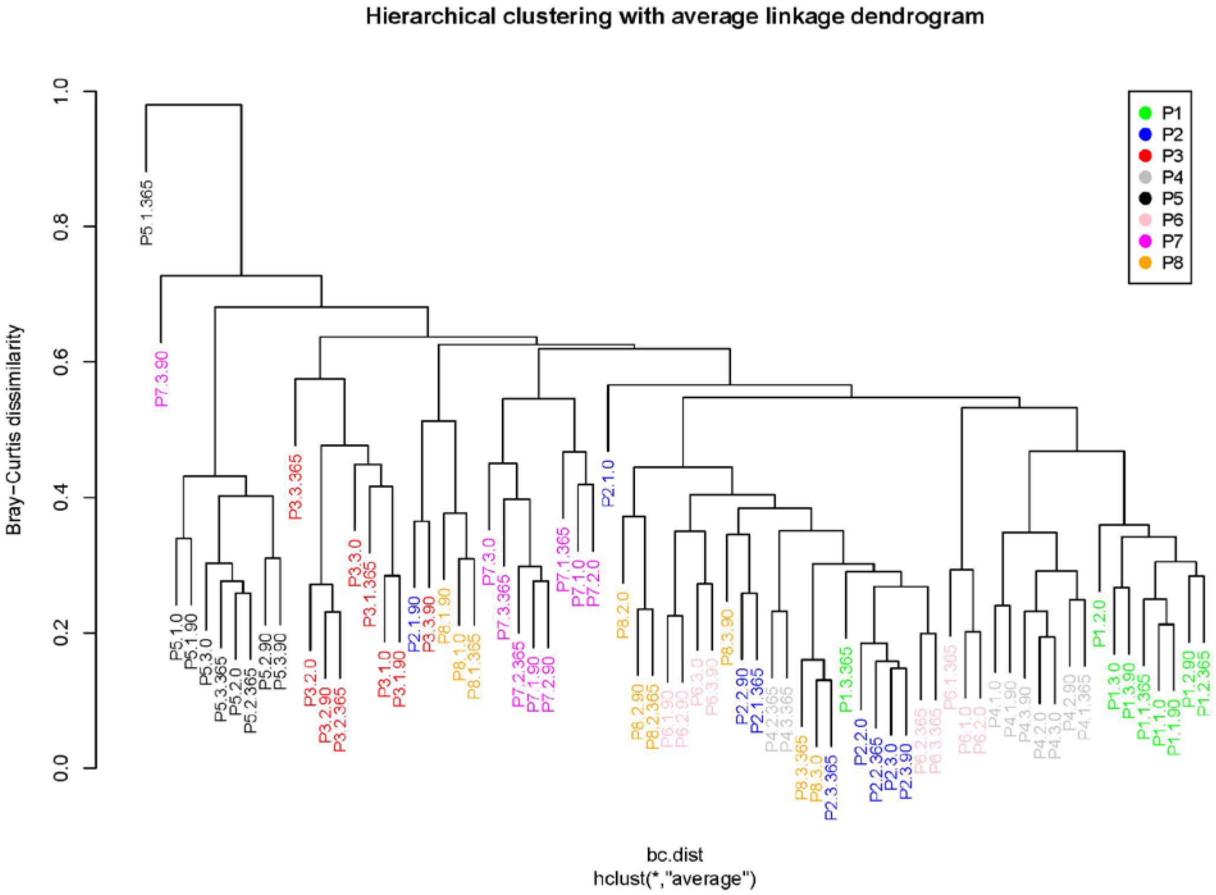

Supplementary Figure 2.
Tree based hierarchical agglomerative clustering with average linkage dendrogram analysis on Bray-Curtis distances with coloring according to volunteer (P1-P8). 1… forearm, 2 … V of the chest and 3 … back. 0…d0, 90…d90, 365…d365.
